# TCF7L2 involvement in estradiol- and progesterone-modulated islet and hepatic glucose homeostasis

**DOI:** 10.1038/srep24859

**Published:** 2016-04-25

**Authors:** Fengqin Dong, Qi Ling, Dan Ye, Zhe Zhang, Jing Shu, Guoping Chen, Yang Fei, Chengjiang Li

**Affiliations:** 1 Department of Endocrinology, First Affiliated Hospital, Zhejiang University School of Medicine, Hangzhou, China; 2 Department of Surgery, Collaborative Innovation Center for Diagnosis and Treatment of Infectious Diseases, First Affiliated Hospital, Zhejiang University School of Medicine, Hangzhou, China; 3 Department of Reproductive Endocrinology, Zhejiang Province People’s Hospital, Hangzhou, China; 4 Department of Metabolism and Endocrinology, People’s Hospital of Fuyang City, Hangzhou, China

## Abstract

To evaluate the role of TCF7L2, a key regulator of glucose homeostasis, in estradiol (E_2_) and progesterone (P_4_)-modulated glucose metabolism, mouse insulinoma cells (MIN6) and human liver cancer cells (hepG2 and HUH7) were treated with physiological concentrations of E_2_ or P_4_ in the up- and down-regulation of TCF7L2. Insulin/proinsulin secretion was measured in MIN6 cells, while glucose uptake and production were evaluated in liver cancer cells. E_2_ increased insulin/proinsulin secretion under both basal and stimulated conditions, whereas P_4_ increased insulin/proinsulin secretion only under glucose-stimulated conditions. An antagonistic effect, possibly concentration-dependent, of E_2_ and P_4_ on the regulation of islet glucose metabolism was observed. After E_2_ or P_4_ treatment, secretion of insulin/proinsulin was positively correlated with TCF7L2 protein expression. When TCF7L2 was silenced, E_2_- or P_4_-promoted insulin/proinsulin secretion was significantly weakened. Under glucotoxicity conditions, overexpression of TCF7L2 increased insulin secretion and processing. In liver cancer cells, E_2_ or P_4_ exposure elevated TCF7L2 expression, enhanced the activity of insulin signaling (pAKT/pGSK), reduced PEPCK expression, subsequently increased insulin-stimulated glucose uptake, and decreased glucose production. Silencing TCF7L2 eliminated effects of E_2_ or P_4_. In conclusion, TCF7L2 regulates E_2_- or P_4_-modulated islet and hepatic glucose metabolism. The results have implications for glucose homeostasis in pregnancy.

Gestational diabetes mellitus (GDM) is seen commonly during pregnancy in predisposed individuals. It is characterized by a combination of reduced maternal insulin secretion and impaired insulin sensitivity. GDM can cause severe maternal and neonatal adverse effects. In the past several decades, the prevalence of GDM has markedly increased worldwide, which is likely due, at last in part, to increased maternal weight^1^,^2^,^3^.

GDM usually develops during the second trimester when estradiol (E_2_) and progesterone (P_4_) levels sharply increase^4^. In predisposed individuals, the production of placental hormones can lead to maternal insulin resistance, which in turn can contribute to the development of GDM. Adapting to insulin resistance, sex hormones can modulate pancreatic islets to adapt to insulin resistance. For instance, E_2_ acts directly on β cells to increase insulin biosynthesis and enhance glucose-stimulated insulin secretion^4^, and also reduces hepatic and muscle insulin resistance^5^. Physiologically elevated P_4_ levels stimulates islet cell proliferation^6^. However, if β-cell functions do not adapt to increased insulin demand, GDM may develop. Other identified risk factors for GDM include advanced maternal age, obesity, ethnicity or race (e.g., Asians), previous history of GDM, and family history of diabetes mellitus^1^.

Genetic background also contributes to development of GDM^7^. Recent systematic reviews reported a significant association between GDM risk and genetic polymorphisms^8^,^9^. Genetic variants of transcription factor 7-like 2 (TCF7L2) demonstrated the strongest association with GDM risk in women of different races and ethnicities^8^. The risk T allele of rs7903146 was associated with increased TCF7L2 protein expression, as well as decreased insulin content and secretion^10^,^11^. TCF7L2, also known as TCF4, is the effector of the Wnt signaling pathway and considered to serve as an important regulator of glucose homeostasis by regulating proinsulin production and processing^10^,^12^. Additionally, TCF7L2 may affect hepatic glucose metabolism by suppressing gluconeogenesis^13^,^14^,^15^,^16^. However, the underlying regulatory properties of TCF7L2 in the development of GDM have not previously been described. In this study, we evaluated E_2_- and P_4_-modulated glucose homeostasis in islets and liver to further assess the physiological relationship between these hormones and TCF7L2.

## Research Design and Methods

### Cell culture and transfection

Human hepatocarcinoma cells (HepG2 and HUH7), mouse insulinoma (MIN6) cells, and 293T cells, obtained from the Chinese Academy of Science, were cultured in DMEM containing fetal calf serum (10%; Hyclone), penicillin (100 U/ml), streptomycin (100 μg/ml), and 1% Glutamax in 5% CO_2_. E_2_ and P_4_ were obtained from Sigma-Aldrich.

HepG2 and MIN6 cells were seeded in a six-well culture plate at a density of 5 × 10^5 ^cells/well. After 24 h, 400 μl of DMEM (serum-free and antibiotic-free) was added with 4 μg of DNA from pcDNA6.2-EGFP-TCF7L2 (full-length TCF7L2, Invitrogen) and pcDNA6.2-EGFP-empty control plasmid or 4 μg of DNA from pcDNA6.2-GW/EGFP-miR-TCF7L2 (NCBI: NM_001146284.1; Supplemental Table 1) and pcDNA6.2-GW/EGFP-miR-NC plasmid, respectively, then mixed thoroughly. Cell suspensions were thoroughly mixed with 9 μl of Lipofectamine™ 2000 transfection reagent and allowed to stand at room temperature for 10 min to form transfection complexes. The complexes were added to the cells and co-cultured at 37 °C in a 5% CO_2_ incubator. The cells were collected at 24–72 h after transfection. The pcDNA6.2- GW/EGFP- miR-TCF7L2 plasmid producing the highest knockdown of TCF7L2 mRNA and protein expression was used for subsequent experiments.

### Cell viability assay

Cell viability was assessed by 3-(4,5-dimethylthiazol-2-yl)-2,5-diphenyltetrazolium bromide (MTT) assay as previously reported^17^. Briefly, cells (1 × 10^5 ^cells/well) were seeded in 96-well plates and incubated overnight. The original medium was replaced with fresh medium containing vehicle control or sex hormones for another 24 h. The MTT solution (2 mg/ml) was added to each well and incubated for 4 h. After centrifugation at 1000 rpm for 10 min, the medium was removed, and the MTT formazan precipitate was dissolved in 100 μl of DMSO. Absorbance was measured at 570 nm with a plate reader (Opsys MR, Denex Technology, USA).

### Proinsulin and insulin release tests

MIN6 cells (5 × 10^5 ^cells/well) were seeded in six-well plates and incubated overnight. Cells were treated with the vehicle control or sex hormones for 24 h. Proinsulin or insulin was released into the medium and measured using the proinsulin or insulin ELISA kit. The proinsulin or insulin secretion was normalized to viable cell numbers.

### Glucose uptake and production assays

Cells (2.5 × 10^5 ^cells/well) seeded in six-well plates were treated with sex hormones for 24 h. The medium was replaced with serum- and glucose-free DMEM for another 4 h. The cells were then washed with PBS and incubated in serum- and glucose-free DMEM containing the fluorescent glucose analog 2-deoxy-2-[(7-nitro-2,1,3-benzoxadiazol- 4-yl)amino]-d-glucose (2-NBDG, 40 μM) and insulin (100 nM) for 30 min. After incubation, cells were washed with PBS, and fluorescence retained by the cells was measured by flow cytometer (BD Bioscience, San Jose, California, USA). Data from 10,000 cells were collected and analyzed.

Cells (5 × 10^4 ^cells/well) seeded in 24-well plates were treated with sex hormones for 24 h. The medium was then replaced with serum- and glucose-free DMEM containing 20 mmol/l d-lactate and 2 mmol/l sodium pyruvate for 20 h. The medium was subsequently assayed for glucose with a commercial kit (Invitrogen). The glucose production was normalized to viable cell numbers.

### Qualitative RT-PCR

Total RNA was extracted using a commercial kit, and real-time PCR was performed with the 9700 Real-time PCR System (Applied Biosystems, Carlsbad, California, USA) and the SDS 2.1 software (Applied Biosystems). The PCR primers are shown in Supplemental Table 2. Each PCR run started with the incubation of samples at 50 °C for 2 min, followed by incubation at 95 °C for 10 min, and 40 cycles of incubation at 95 °C for 15 s and at 59 °C for 30 s. Data were normalized by quantifying the amount of amplified cDNA products by calculating the ratio of the amount of cDNA relative to the amount of GAPDH cDNA.

### Western blot analysis

Western blot analysis was performed as previously reported^17^. The antibodies previously used for Western blots were the anti-human TCF7L2 antibody (1:800; 13838-1-AP, Proteintech, USA), anti-human PEPCK antibody (1:1000; 14892-1-AP; Proteintech, USA), anti-human GLUT2 antibody (1:800; 20436-1-AP; Proteintech, USA), anti-human IRS2 antibody (1:1000; 20702-1-AP; Proteintech, USA), anti-human pAKT antibody (1:1000; 60072-1-Ig; Proteintech, USA), anti-human AKT antibody (1:1000; 10176-2-AP; Proteintech, USA), anti-human pGSK antibody (1:1000; 14850-1-AP; Proteintech, USA), anti-human GSK antibody (1:1000; 22104-1-AP; Proteintech, USA), anti-human pERK1/2 antibody (1:1000; 3441-100; BioVision, USA), anti-human ERK1/2 antibody (1:1000; 16443-1-AP; Proteintech, USA), and anti-human GAPDH antibody (1:1000; 10494-1-AP; Proteintech, USA). GAPDH was used as control. The results were quantified by the ImagePro Plus 5.1 software (Media Cybernetics, Inc., USA).

### Statistical analysis

Statistical analyses were performed with SPSS for Windows version 13.0 (SPSS, Inc., Chicago, USA). Values with P < 0.05 were considered statistically significant. The Kolmogorov–Smirnov test was used to check the normality. Quantitative variables were expressed as mean ± SD or median. Categorical variables were presented as values and percentages. One-way ANOVA with Dunnett’s t-test, Student’s t-test, Wilcoxon–Man–Whitney test, and chi-square test were used to compare the variables. Pearson correlation test was used for correlation analysis.

## Results

### Effects of estradiol and progesterone on β-cell survival and function

**Figure 1:**
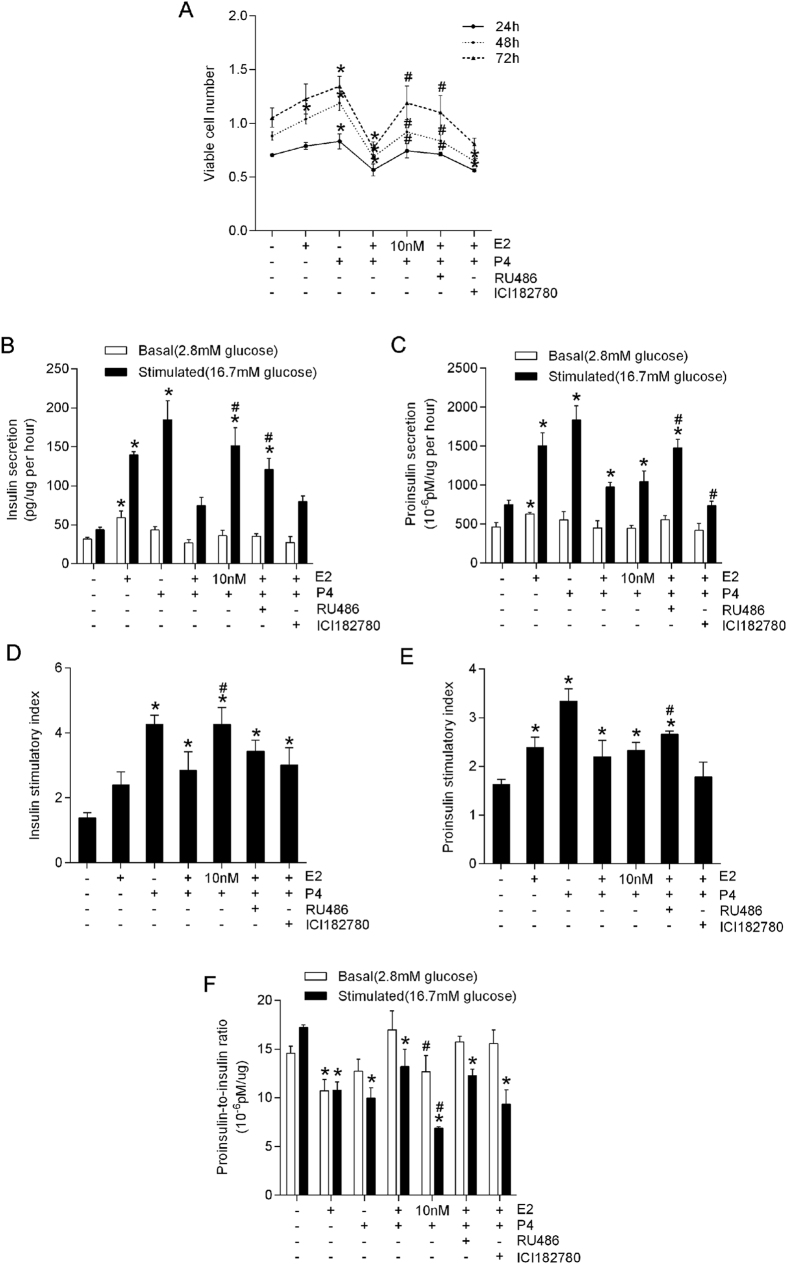
Effects of estradiol (E_2_) and progesterone (P_4_) on β-cell survival and function. MIN6 cells were plated at 5 × 10^5^ cells per well in 6-well plates and treated with different combination of E_2_ (10 or 100 nM), P_4_ (1 μM), progesterone receptor antagonist (RU486 40 uM) and estradiol receptor antagonist (ICI182780 10 uM) for 24 h. The concentrations of hormones are in the physiological ranges. (**A**) Viable cells. (**B,C**) Basal and stimulated insulin/proinsulin secretions (normalized to viable cell numbers). (**D,E**) Stimulatory indexes. (**F**) Proinsulin-to-insulin ratio. ^*^P < 0.05 vs. blank control; ^#^*P* < 0.05 vs. E_2_ (100 nM) plus P_4_ (1 μM).

To evaluate the effects of E_2_ and P_4_ on β-cell proliferation, we treated MIN6 cells with 100 nM E_2_, 1 μM P_4_, or 100 nM E_2_ plus 1 μM P_4_. We found that the sole use of E_2_ or P_4_ increased the number of viable cells (Fig. 1A), but E_2_ and P_4_ together significantly decreased the number of viable cells at 24, 48, and 72 h (Fig. 1A, *P* < 0.05).

E_2_ can stimulate progesterone receptor (PR) activity^18^. Overactivation of P_4_/PR signaling exerted an unfavorable effect on β-cell survival and function^19^,^20^,^21^. Thus, we further exposed the cells to a low concentration of E_2_ (10 nM) plus P_4_ and found a significant increase in the number of viable cells compared with those treated with high concentration of E_2_ (100 nM) plus P_4_ (Fig. 1A, *P* < 0.05). A similar result was observed after PR was blocked with antagonist RU486 (40 μM) but not after ER was blocked with ICI182780 (10 μM) (Fig. 1A).

Insulin and proinsulin secretion were evaluated after sex hormone treatment in MIN6 cells. When the cells were cultured in E_2_ alone for 24 h, they exhibited significantly increased insulin/proinsulin secretion under basal and glucose-stimulated conditions (Fig. 1B,C, *P* < 0.05) compared with the control cells. Cells treated with P_4_ alone showed significantly increased insulin/proinsulin secretion only under glucose-stimulated conditions (Fig. 1B,C, *P* < 0.05). However, co-treatment of E_2_ and P_4_ demonstrated a much weaker effect on insulin/proinsulin secretion than the effect caused by E_2_ or P_4_ treatment alone. After reducing the concentration of E_2_ or blocking PR, cells treated with E_2_ plus P_4_ showed significantly increased insulin/proinsulin secretion (Fig. 1B,C, *P* < 0.05). The insulin/proinsulin stimulatory index and insulin processing capability (reflected by the proinsulin-to-insulin ratio) followed the same tendency as the insulin/proinsulin secretory dynamic (Fig. 1D–F).

### Silencing of TCF7L2 weakens estradiol- and progesterone-promoted β-cell insulin secretion

**Figure 2:**
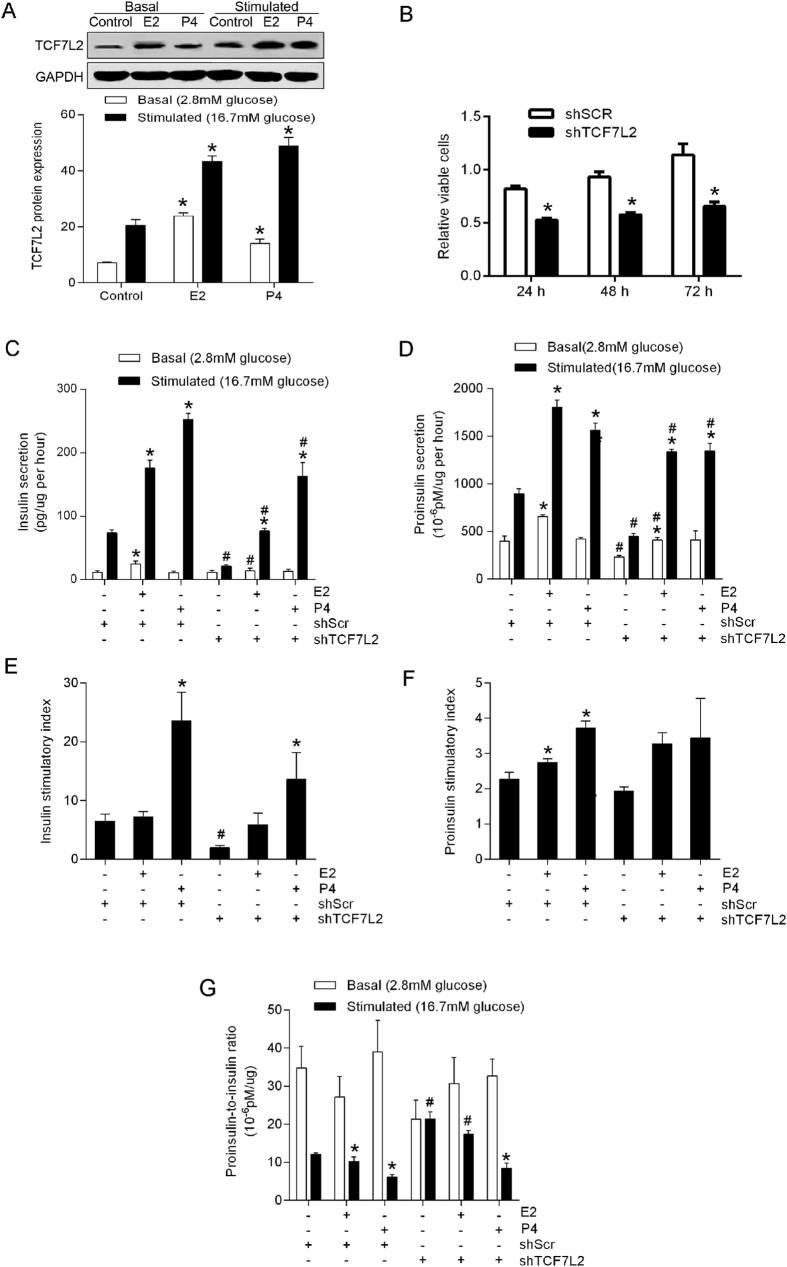
Silencing of TCF7L2 reduces estradiol (E_2_)- and progesterone (P_4_)-promoted β-cell insulin/proinsulin secretion. MIN6 cells were plated at 5 × 10^5 ^cells per well in 6-well plates and exposed to a TCF7L2-specific short hairpin RNA (shTCF7L2) or a scrambled shRNA (shScr) for 72 h, then cultured for 24 h in the presence of 100 nM E_2_ or 1 μM P_4_. (**A**) Western blots showing the TCF7L2 protein content after E_2_ or P_4_ treatment. (**B**) Viable cells. (**C,D**) Basal and stimulated insulin/proinsulin secretions (normalized to viable cell numbers). (**E,F**) Stimulatory indexes. (**G**) Proinsulin-to-insulin ratio. ^*^P < 0.05 vs. sex hormone treatment control; ^#^*P* < 0.05 shTCF7L2 vs. shScr.

To assess the role of TCF7L2 in E_2_- or P_4_-modulated β-cell function, we analyzed the TCF7L2 protein content after sex hormone exposure. E_2_ markedly increased TCF7L2 protein content under both basal and stimulated conditions, whereas P_4_ sharply increased TCF7L2 protein content under stimulated conditions (Fig. 2A). Similar effect was found in TCF7L2 mRNA levels after sex hormone exposure (Supplemental Fig. 1). The TCF7L2 protein content showed a significantly positive correlation with the insulin/proinsulin secretion after E_2_ or P_4_ treatment (Supplemental Table 3; Figs 1 and 2).

We knocked down TCF7L2 in MIN6 cells and found that the number of viable cells significantly decreased at 24, 48, and 72 h (Fig. 2B, *P* < 0.05). Decreased TCF7L2 expression led to significantly lower insulin/proinsulin secretion (Fig. 2C,D, *P* < 0.05). More importantly, the downregulation of TCF7L2 markedly weakened the E_2_- or P_4_-promoted insulin/proinsulin secretion (Fig. 2C,D, *P* < 0.05). E_2_ did not significantly change the insulin stimulatory index, whereas P_4_ remarkably increased insulin and proinsulin stimulatory indexes (Fig. 2E,F). The downregulation of TCF7L2 weaken the P_4_-elevated insulin/proinsulin stimulatory index (Fig. 2E,F). Furthermore, P_4_-promoted insulin processing persisted after the downregulation of TCF7L2, as reflected by the significantly decreased proinsulin-to-insulin ratio compared with that of the control (Fig. 2G, *P* < 0.05).

### Overexpression of TCF7L2 increases insulin secretion and processing under glucotoxicity status

To mimic diabetic glucotoxicity, we exposed MIN6 cells to a high glucose concentration (33.3 mM) for 72 h. Although an increase in basal secretion of insulin was observed, we found significantly reduced insulin secretion and increased proinsulin-to-insulin ratio at stimulated conditions in high glucose-exposed MIN6 cells compared with 5.5 mM glucose-cultured cells (*P* < 0.05), thereby indicating impaired glucose-stimulated insulin secretion and processing capability in the setting of glucotoxicity.

**Figure 3:**
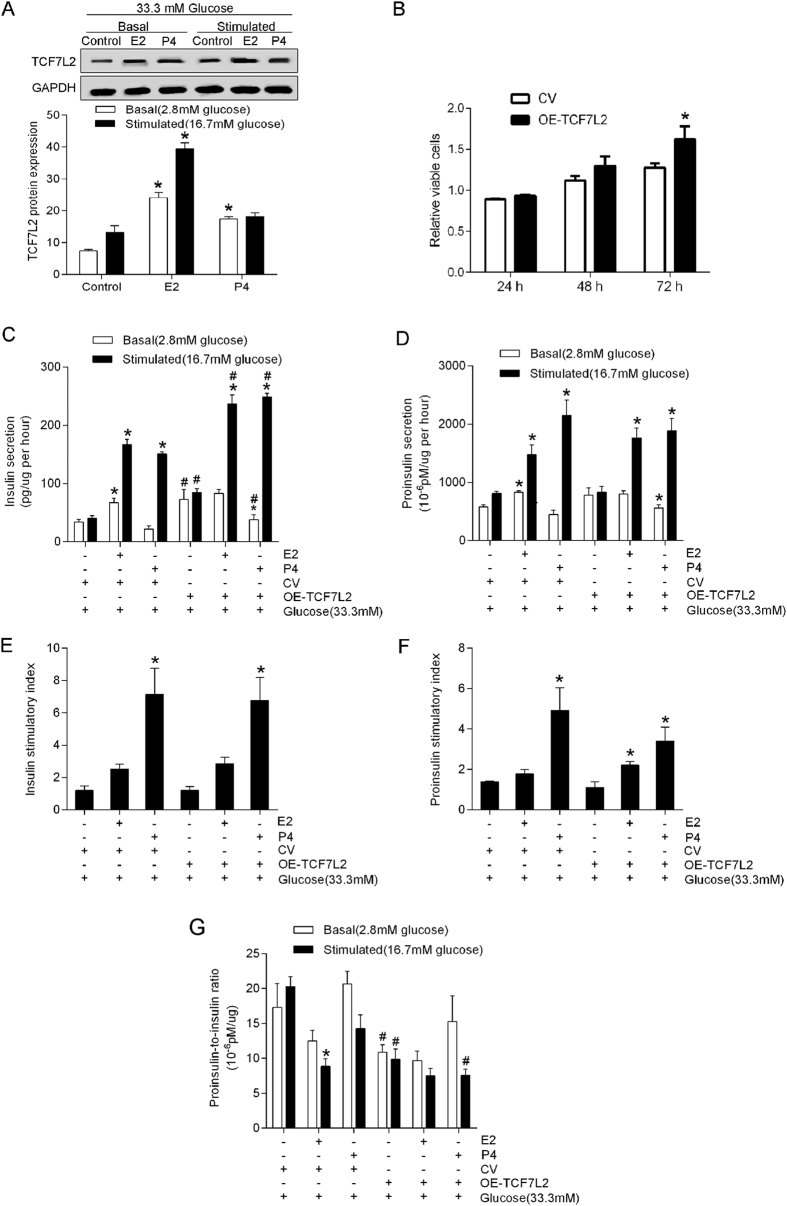
Overexpression of TCF7L2 increases insulin processing under glucotoxicity status. MIN6 cells were plated at 5 × 10^5^ cells per well in 6-well plates and exposed to high glucose concentration (33.3 mM) and transfected with TCF7L2-IRES2-EGFP (OE-TCF7L2) or a control vector (CV) for 72 h, then cultured for 24 h in the presence of 100 nM E_2_ or 1 μM P_4_. (**A**) Western blots showing the TCF7L2 protein content after E_2_ or P_4_ treatment. (**B**) Viable cells. (**C**,**D**) Basal and stimulated insulin/proinsulin secretions (normalized to viable cell numbers). (**E,F**) Stimulatory indexes. (**G**) Proinsulin-to-insulin ratio. ^*^P < 0.05 vs. sex hormone treatment control; ^#^*P* < 0.05 OE-TCF7L2 vs. CV.

Under glucotoxicity conditions, we found that P_4_ cannot stimulate TCF7L2 protein content (Fig. 3A) and cannot reduce the proinsulin-to-insulin ratio under stimulated condition (Fig. 3G). By contrast, E_2_ treatment was associated with similar effects on insulin and proinsulin secretion as under normal conditions (Figs 2 and 3)

Overexpression of TCF7L2, led to increased number of viable cells (Fig. 3B), significantly increased basal and stimulated insulin secretions (Fig. 3C, *P* < 0.05) and enhanced insulin processing capability, as reflected by a significantly decreased proinsulin-to-insulin ratio under glucotoxicity conditions (Fig. 3G, *P* < 0.05). However, the stimulatory indexes remained unchanged (Fig. 3E,F, *P *> 0.05).

### TCF7L2 participates in estradiol- and progesterone-regulated hepatic glucose metabolism

**Figure 4:**
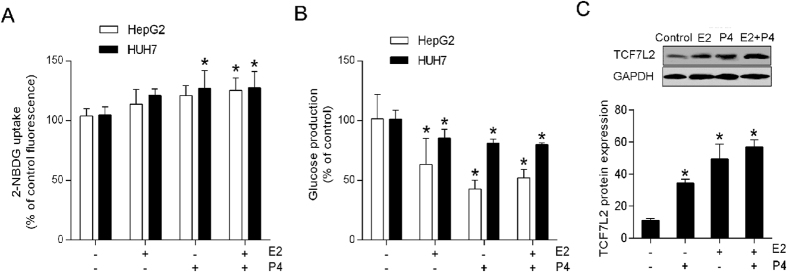
Estradiol (E_2_) and progesterone (P_4_) regulate hepatic glucose metabolism. HepG2 and HUH7 cells (2.5 × 10^5 ^cells per well) were seeded in 6-well plates and treated with 100 nM E_2_, 1 μM P_4_, or E_2_ plus P_4_ for 24 h. (**A**) 2-NBDG uptake. (**B**) Glucose production. (**C**) Western blot. ^*^P < 0.05 vs. sex hormone treatment control.

Since the liver plays a central role in controlling glucose uptake and production, we assessed the effect of sex hormones on hepatic glucose metabolism. We treated hepG2 and HUH7 cells with 100 nM E_2_, 1 μM P_4_, or 100 nM E_2_ plus 1 μM P_4_. The numbers of viable cells were similar in each sex hormone-treated group compared with the controls at 24, 48, and 72 h (P > 0.05). We also found a significantly increased insulin-stimulated glucose uptake and decreased glucose production after E_2_, P_4_, or combined treatment (Fig. 4A,B). Moreover, TCF7L2 protein content markedly increased after E_2_, P_4_, or combined treatment (Fig. 4C).

**Figure 5:**
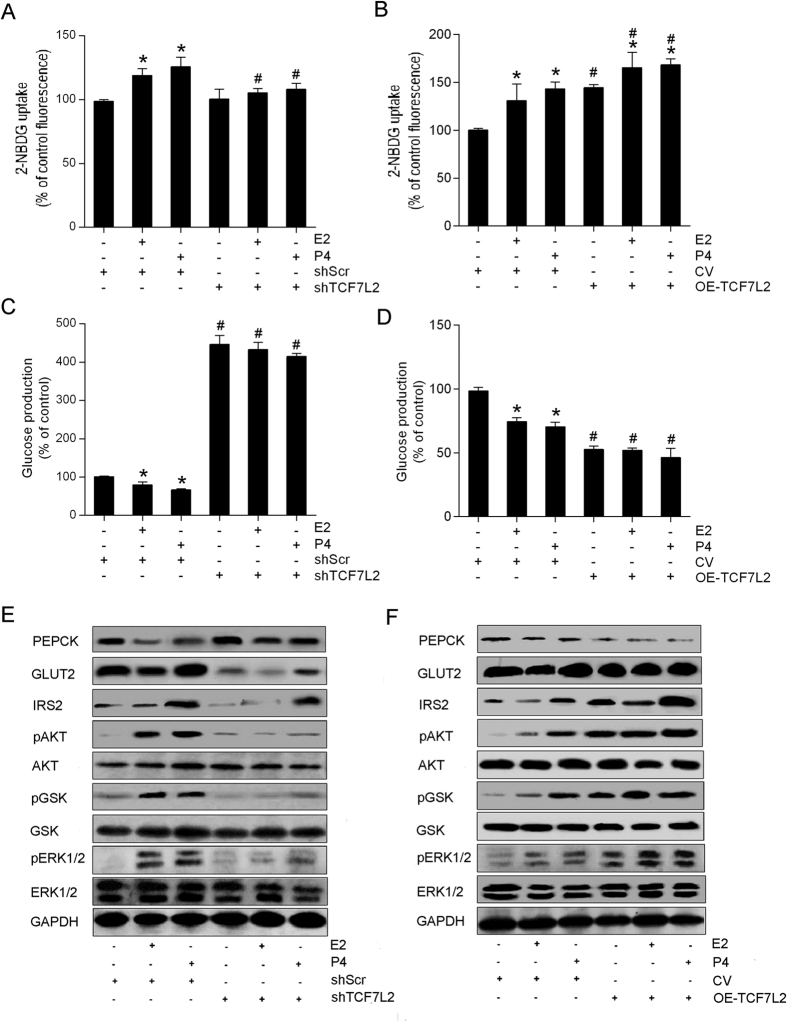
TCF7L2 involves in estradiol (E_2_)- and progesterone (P_4_)-regulated hepatic glucose metabolism. HepG2 cells (2.5 × 10^5^ cells per well) were seeded in 6-well plates and exposed to a TCF7L2-specific short hairpin RNA (shTCF7L2) or a scrambled shRNA (shScr) for 72 h, or transfected with TCF7L2-IRES2-EGFP (OE-TCF7L2) or a control vector (CV) for 72 h, then cultured for 24 h in the presence of 100 nM E_2_ or 1 μM P_4_. (**A,B**) 2-NBDG uptake. (**C,D**) Glucose production. (**E,F**) Western blot. ^*^P < 0.05 vs. sex hormone treatment control; ^#^*P* < 0.05 shTCF7L2 vs. shScr or OE-TCF7L2 vs. CV.

After TCF7L2 downregulation in hepG2 cells, the effect caused by E_2_ or P_4_ disappeared (Fig. 5A,C). Overexpression of TCF7L2 increased insulin-stimulated glucose uptake and decreased glucose production in cells with or without sex hormone treatment (Fig. 5B,D).

To further investigate the mechanism of sex hormone-modulated hepatic glucose metabolism, we examined the expression of several key molecules after E_2_ or P_4_ treatment. We found that both E_2_ and P_4_ reduced the expression of PEPCK but increased the expression of pAKT, pGSK, and pERK1/2 proteins (Fig. 5E). By silencing TCF7L2, the effect of E_2_- and P_4_-stimulated phosphorylation of AKT, GSK, and ERK1/2 was significantly weakened. However, P_4_ induced the elevated IRS2 expression in the presence and absence of TCF7L2. Additionally, silencing of TCF7L2 increased the PEPCK expression but decreased the expression of GLUT2 and IRS2 in each group (Fig. 5E), whereas overexpression of TCF7L2 produced the opposite effect (Fig. 5F). The quantification of western blots is presented in Supplemental Fig. 2. We also found the same effects of E_2_ and P_4_ in mRNA levels of PEPCK, GLUT2, and IRS2 (Supplemental Fig. 3).

## Discussion

The increased levels of estrogenic and progestational hormones can generally lead to insulin resistance and elevated glucose levels. However, pregnant women were found to exhibit lower blood glucose levels than those of non-pregnant ones^22^. Updated guidelines recommend lower blood glucose values to diagnose GDM^22^.

E_2_ and P_4_ induce opposing physiological effects on the female reproductive system and other organ systems, such as the cardiovascular system^22^. The present study demonstrates that the two sex hormones may exert an antagonistic effect on regulating islet glucose homeostasis. Our results reveal that acute treatment of E_2_ or P_4_ leads to elevated β-cell proliferation, increased insulin/proinsulin production. This response seems to be an adaptation of islets to maintain maternal glucose homeostasis, even under conditions of glucotoxicity. However, combined E_2_ and P_4_ exposure resulted in an opposite effect, namely decreased cell proliferation and reduced glucose-stimulated responsivity and insulin processing. A previous report implied that the antagonistic effect permitted P_4_ to stimulate islet cell proliferation, but the effect was suppressed by E_2_ supplementation^6^. However, the sole use of E_2_ did not affect β-cell proliferation^23^ but prevented apoptosis under oxidative stress injury^24^.

To explain this series of events, it is necessary to focus first on the paradoxical effect of P_4_ on β cells. Previous studies have shown that the physiological concentration of P_4_ stimulates β-cell proliferation^6^ and increases insulin secretion under conditions of oxidative stress^25^. By contrast, pharmacological or supra-physiological concentrations of P_4_ will exert an unfavorable effect on β-cell survival and function^21^,^26^. Overstimulation of P_4_/PR signaling will lead to reduced β-cell viability^19^,^20^. E_2_ can markedly stimulate PR activity in β cells^18^; thus, we assumed that E_2_ may reinforce P_4_/PR signaling and cause an amplification effect. As expected, we found that the antagonistic effect can be significantly attenuated by reducing the concentration of E_2_ during cotreatment or by inhibiting the PR. By contrast, the ER antagonist cannot achieve such an effect because it inhibits the E_2_ signal but, moreover, cannot influence overstimulated P_4_/PR signaling. Overall, our results suggest a role for the interplay between sex hormones and the development of GDM. However, further studies, are with regard to this observation.

TCF7L2 is the most powerful, recognized susceptibility gene for diabetes. The association of TCF7L2 gene polymorphisms with almost all subtypes of diabetes (e.g., type 1 and 2 diabetes, latent autoimmune diabetes in adults, hepatogenous diabetes, and post-transplant diabetes) has been demonstrated^27^,^28^,^29^,^30^. Zhou *et al.* proposed a master role for TCF7L2 in regulating insulin biosynthesis, secretion, and processing^10^. But evidence from Boj *et al.* was less convincing^14^. Our results revealed that TCF7L2 was essential for glucose-stimulated insulin/proinsulin secretion and processing, as well as β-cell proliferation. Furthermore, under conditions of glucotoxicity, the overexpression of TCF7L2 will markedly increase insulin processing and secretion. These findings attest to the significant role of TCF7L2 in islet function and indicate a potential clinical role of TCF7L2 in treating diabetes.

We further found a positive correlation between E_2_- or P_4_-increased TCF7L2 protein content and E_2_- or P_4_-promoted insulin/proinsulin secretion. Downregulation of TCF7L2 remarkably weakened the effect induced by E_2_ or P_4_, thereby indicating that E_2_- or P_4_-promoted insulin/proinsulin secretion can, at least in part, be TCF7L2 dependent. Moreover, the E_2_-promoted stimulated insulin processing is TCF7L2 required; whereas the P_4_- promoted stimulated insulin processing may be independent of TCF7L2 because the effect persisted in the absence of TCF7L2 expression. In addition, TCF7L2 may not be essential for the E_2_- or P_4_-increased stimulatory index since downregulation of TCF7L2 did not alter the effects in stimulatory index. Under conditions of glucotoxicity, E_2_ increased TCF7L2 protein content under basal and stimulated conditions as it did under the normal, non-glucotoxic conditions to achieve similar effects on β-cell secretion. In contrast, P_4_ did not further increase TCF7L2 protein content under stimulated conditions. Thus, insulin processing was unaffected. However, P_4_ still induced enhanced insulin/proinsulin secretion under stimulated conditions. The results suggest that TCF7L2 is essential for insulin processing. However, TCF7L2 was not required for P_4_-promoted stimulated insulin/proinsulin secretion in β cells that were glucotoxic.

Given the central role of the liver in the regulation of glucose homeostasis, we also assessed the effects of E_2_ and P_4_ on hepatic glucose metabolism. Although the opposing effects of E_2_ and P_4_ on oxidative stress processes have been reported *in vitro*^31^,^32^,^33^, a recent *in vivo* study using microarray analysis showed that E_2_, P_4_, and their co-treatment actually displayed similar patterns and networks of hepatic gene expression^34^. Consistent with the previous study, we found that E_2_ and P_4_ presented similar phenotypes and protein content profiles in regulating hepatic glucose metabolism. Both E_2_ and P_4_ can improve insulin-stimulated glucose uptake, possibly through the enhanced activation of hepatic insulin signaling (pAKT/pGSK). E_2_ and P_4_ can also reduce gluconeogenesis by repressing PEPCK expression. Both E_2_ and P_4_ significantly increased hepatic ERK activation, which can increase glycogen synthesis and attenuate glucose output^35^. In addition, the opposing effects of P_4_ and E_2_ were detected in the expression of IRS2 and GLUT2, which may further explain the stronger effect of P_4_ than E_2_ in regulating hepatic glucose uptake.

Combined with previous studies, our study has demonstrated that TCF7L2 represses hepatic glucose production by regulating gluconeogenic genes^13^,^14^,^15^,^16^. In addition, we revealed that TCF7L2 mediated hepatic insulin signaling (IRS2-AKT-GSK), glucose sensing (GLUT2), and energy metabolism (ERK). Norton *et al.* have revealed that TCF7L2 binds directly to multiple genes that are important in hepatic glucose metabolism (e.g., PCK1, IRS2, AKT2 and PDK4) using CHIP-Seq *in vitro*^36^. Therefore, TCF7L2 may modulate hepatic glucose homeostasis in various ways far more than gluconeogenesis^37^. Moreover, this study demonstrates that TCF7L2 is involved in E2- or P_4_-modulated liver glucose metabolism. E_2_ or P_4_-stimulated activation of AKT/GSK and ERK1/2 signaling seems to be TCF7L2-dependent because silencing of TCF7L2 clearly reverses sex hormone-induced phosphorylation of AKT/GSK and ERK1/2 proteins. In contrast, the P_4_-induced overexpression of IRS2 may be TCF7L2-independent because the effect did not diminish after the silencing of TCF7L2.

This study has several limitations. First, the interplay between different concentrations of E_2_ and P_4_ is complex. We did not assess the correlation between TCF7L2 and E_2_ plus P_4_. The genomic and non-genomic mechanisms of the antagonistic effect need further research in future well-designed studies. Second, this work is an *in vitro* study with short-term sex hormone treatment. Thus, *in vivo* studies are needed to further document our findings.

In conclusion, both E_2_ and P_4_ at physiological concentrations modulate glucose homeostasis to meet pregnancy demands, including increased insulin secretion and processing, enhanced hepatic insulin signaling, and reduced gluconeogenesis. The reported insulin resistance caused by E_2_ or P_4_ may be attributable to other mechanisms, such as peripheral insulin resistance and insulin binding^38^,^39^. The antagonistic effect of E_2_ and P_4_ on the regulation of islet glucose metabolism, possibly concentration-dependent, was also observed. Therefore, pregnant women with different combinatorial amounts of sex hormones may be at differential risk for developing GDM. Finally, TCF7L2 is an important regulator of both islet and hepatic glucose metabolism. TCF7L2 participates in E_2_- or P_4_-modulated glucose homeostasis, particularly in hepatic glucose production. Compared with P_4_, E_2_ seems to be more dependent upon expression of TCF7L2 to regulate glucose metabolism.

## Additional Information

**How to cite this article**: Dong, F. *et al.* TCF7L2 involvement in estradiol- and progesterone-modulated islet and hepatic glucose homeostasis. *Sci. Rep.* **6**, 24859; doi: 10.1038/srep24859 (2016).

## Supplementary Material

Supplementary Dataset 1
