# Supplementary material for: TCF7L2 involvement in estradiol- and progesterone-modulated islet and hepatic glucose homeostasis
Source: Sci Rep. 2016 Apr 25;6:24859. doi: 10.1038/srep24859 (PMC4876840; doi:10.1038/srep24859)

**TCF7L2 involvement in estradiol- and progesterone-modulated islet and hepatic glucose homeostasis**

Fengqin Dong ^1^, Qi Ling ^2^, Dan Ye ^1^, Zhe Zhang ^1^, Jing Shu ^3^, Guoping Chen ^1^, Yang Fei ^4^, Chengjiang Li ^1^

^1^ Department of Endocrinology, First Affiliated Hospital, Zhejiang University School of Medicine, Hangzhou, China

^2^ Department of Surgery, Collaborative Innovation Center for Diagnosis and Treatment of Infectious Diseases, First Affiliated Hospital, Zhejiang University School of Medicine, Hangzhou, China

^3^ Department of Reproductive Endocrinology, Zhejiang Province People’s Hospital, Hangzhou, China

^4^ Department of Metabolism and Endocrinology, People’s Hospital of Fuyang City, Hangzhou, China

**Correspondence**:

Qi Ling, PhD, MD

Department of Surgery, First Affiliated Hospital, Zhejiang University School of Medicine

79 Qingchun Road, Hangzhou, 310003, China

Supplemental Table 1. Designed four pair of inference miRNA oligo-specific for TCF7L2 gene sequence

| **Name** | **Sequence** |
| --- | --- |
| miR1-F | 5′- TGCTGAAATCCGGAGGAACGGAGTAGGTTTTGGCCACTGACTGACCTACTCCGCCTCCGGATTT -3′ |
| miR1-R | 5′- CCTGAAATCCGGAGGCGGAGTAGGTCAGTCAGTGGCCAAAACCTACTCCGTTCCTCCGGATTTC -3′ |
| miR2-F | 5′- TGCTGTGATTGGGTACACTGGTTGACGTTTTGGCCACTGACTGACGTCAACCAGTACCCAATCA -3′ |
| miR2-R | 5′- CCTGTGATTGGGTACTGGTTGACGTCAGTCAGTGGCCAAAACGTCAACCAGTGTACCCAATCAC -3′ |
| miR3-F | 5′- TGCTGTATGGAGTGAGCCGACATCACGTTTTGGCCACTGACTGACGTGATGTCCTCACTCCATA -3′ |
| miR3-R | 5′- CCTGTATGGAGTGAGGACATCACGTCAGTCAGTGGCCAAAACGTGATGTCGGCTCACTCCATAC -3′ |
| miR4-F | 5′- TGCTGATATGAAGCTGTCGCTCCTTCGTTTTGGCCACTGACTGACGAAGGAGCCAGCTTCATAT -3′ |
| miR4-R | 5′- CCTGATATGAAGCTGGCTCCTTCGTCAGTCAGTGGCCAAAACGAAGGAGCGACAGCTTCATATC -3′ |
| miR-F  (mouse) | 5'- TGCTGGAATGCATTAAGGGGCTTCTTGTTTTGGCCACTGACTGACAAGAAGCCTTAATGCATTC -3' |
| miR-R  (mouse) | 5'- CCTGGAATGCATTAAGGCTTCTTGTCAGTCAGTGGCCAAAACAAGAAGCCCCTTAATGCATTCC-3' |

**Supplemental Table 2. PCR primers**

| TCF7L2 | TCF7L2-F: 5'-CCCCTGACTTGAACCCACCC-3' |
| --- | --- |
|  | TCF7L2-R: 5'-CCCTCGTCGTCGGATTTGAT-3' |
| PEPCK | PEPCK-F: 5'-TGTCGCTCCTGGGACTTCA-3' |
|  | PEPCK-R: 5'-CTCGGCCACATTGGTAAAGATT-3' |
| GLUT2 | GLUT2-F: 5'-AATTGCTCCAACCGCTCTCA-3' |
|  | GLUT2-R: 5'-CTAATAAGAATGCCCGTGACGAT-3' |
| IRS2 | IRS2-F: 5'- GGCTTCCAGAATGGTCTCAA-3' |
|  | IRS2-R: 5'- AAGTCAATGCTGGCGTAGGT-3' |
| GAPDH | GAPDH-F: 5'-GTCGGTGTGAACGGATTTG-3' |
|  | GAPDH-R: 5'-TCCCATTCTCAGCCTTGAC-3' |

Supplemental Table 3. Correlation between TCF7L2 protein expression and insulin/proinsulin secretion after E_2_ or P_4_ treatment

| Treatment | Condition | Secretion | *P* value | *r* |
| --- | --- | --- | --- | --- |
| E_2_ | Basal | Insulin | 0.003 | 0.956 |
|  |  | Proinsulin | <0.001 | 0.994 |
|  | Stimulated | Insulin | 0.013 | 0.904 |
|  |  | Proinsulin | 0.001 | 0.974 |
| P_4_ | Basal | Insulin | 0.009 | 0.921 |
|  |  | Proinsulin | 0.001 | 0.969 |
|  | Stimulated | Insulin | 0.266 | 0.542 |
|  |  | Proinsulin | <0.001 | 0.987 |

Pearson correlation test was used for analysis.

Supplemental Figure 1. The effect on estradiol (E_2_) and progesterone (P_4_) on β-cell TCF7L2 mRNA levels. MIN6 cells were plated at 5×10^5^ cells per well in 6-well plates and cultured for 24 h in the presence of 100 nM E_2_ or 1 µM P_4_. ^*^: *P* < 0.05.


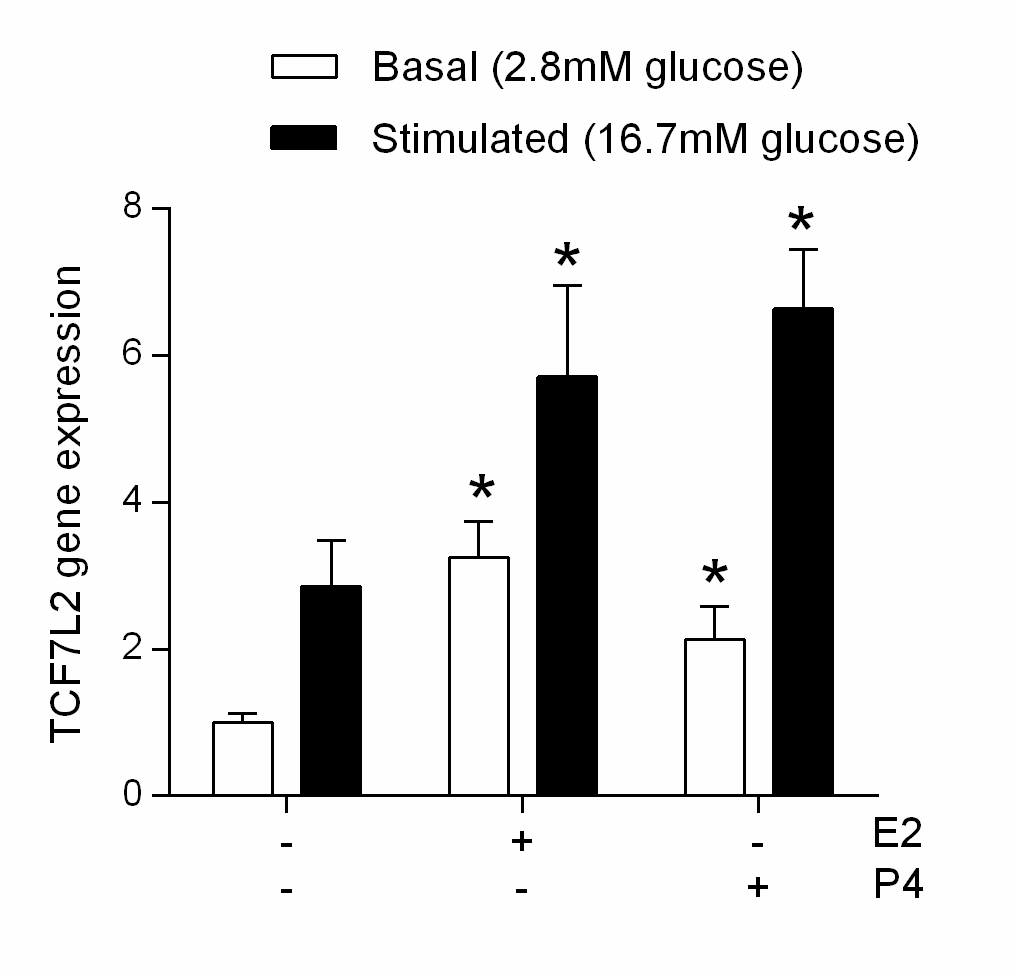


Supplemental Figure 2. The quantification of western blots in Figure 5E and 5F. HepG2 cells (2.5×10^5^ cells per well) were seeded in 6-well plates and exposed to a TCF7L2-specific short hairpin RNA (shTCF7L2) or a scrambled shRNA (shScr) for 72 h, or transfected with TCF7L2-IRES2-EGFP (OE-TCF7L2) or a control vector (CV) for 72 h, then cultured for 24 h in the presence of 100 nM E_2_ or 1 µM P_4_. ^*^: *P* < 0.05 vs. sex hormone treatment control; ^#^: *P* < 0.05 shTCF7L2 vs. shScr, or OE-TCF7L2 vs. CV.

Supplemental Figure 3. The effects of estradiol (E_2_) and progesterone (P_4_) on hepatic PEPCK, GLUT2, and IRS2 mRNA levels. HepG2 cells (2.5×10^5^ cells per well) were seeded in 6-well plates and cultured for 24 h in the presence of 100 nM E_2_ or 1 µM P_4_. ^*^: *P* < 0.05.


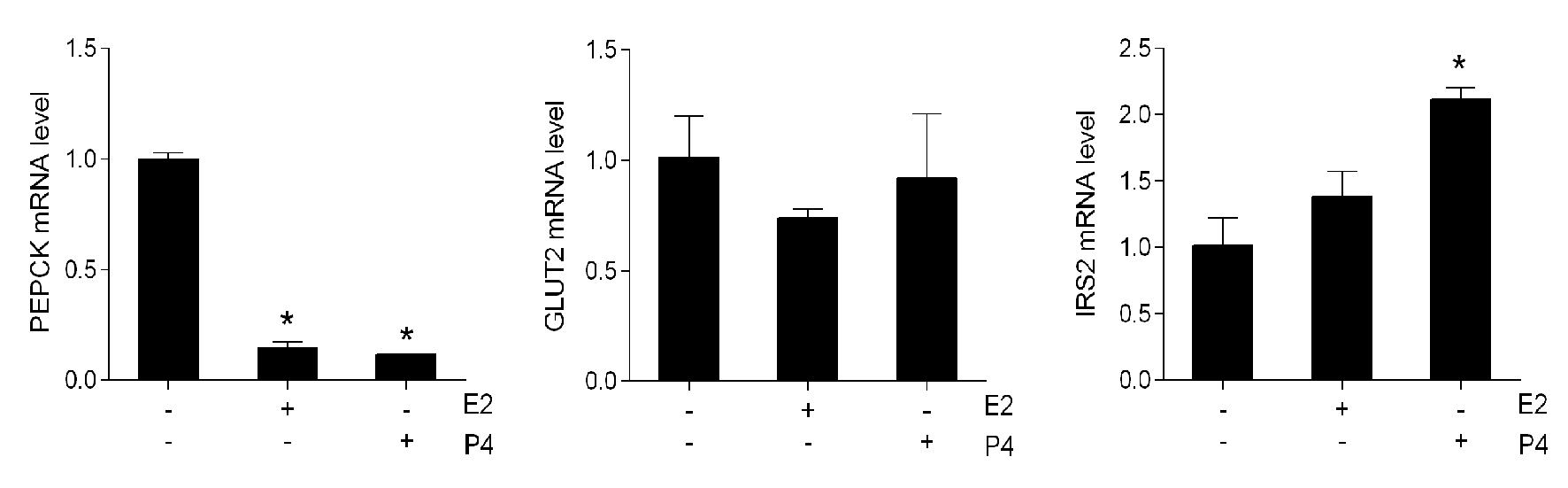

Supplement: Supplementary Dataset 1 [file srep24859-s1.doc]
